# Imaging findings of penetrating spinal cord injuries secondary to stab wounds on magnetic resonance imaging in a tertiary trauma unit, South Africa

**DOI:** 10.4102/sajr.v23i1.1761

**Published:** 2019-09-19

**Authors:** Jacolien M. Rall, Fekade A. Gebremariam, Gina Joubert

**Affiliations:** 1 Department of Clinical Imaging Sciences, University of the Free State, Bloemfontein, South Africa; 2 Department of Biostatistics, University of the Free State, Bloemfontein, South Africa

**Keywords:** Spinal cord stab wounds, MRI findings, neurological performance, trauma, infection

## Abstract

**Background:**

In South Africa, the leading cause of spinal cord injuries is motor vehicle accidents, followed by violence-related injuries, including gunshot injuries and stab wounds. Controversy regarding management persists. Magnetic resonance imaging (MRI) is the gold standard to rule out surgical causes of neurological deficit.

**Objectives:**

To determine the spectrum of imaging findings in penetrating spinal cord injuries, specifically related to stab wounds, in a Tertiary Academic Hospital in the Free State province and whether these imaging findings influenced immediate surgical decision-making and outcomes of patients.

**Method:**

Consecutive sampling was used to retrospectively select patients who presented with spinal penetrating injuries secondary to stab wounds during the period 01 August 2013–30 September 2016 and received MRI investigation. Fifty-six patients were included. Magnetic resonance imaging investigations were reviewed by the authors, with documentation of MRI findings, relevant patient demographics and clinical information into Excel spread sheets. Statistical analysis was performed by the Biostatistics Department of the University of the Free State.

**Results:**

The most common MRI finding was a high signal intensity wound tract (96.6%), followed by cord signal changes (91.1%) and cord oedema (82.1%). Thirty-nine extra-axial collections were diagnosed in 30 penetrating injuries, of which only one had spinal compressive effects. Four patients (7.1%) demonstrated pseudo-meningoceles. None of the included patients had an indication for emergency spinal surgery on review of imaging.

**Conclusion:**

Magnetic resonance imaging findings did not alter the surgical course of action in our study patients. Despite this, MRI is a valuable modality in evaluation of penetrating spinal cord injuries in the post-traumatic phase (<24 h) for the presence of pseudo-meningoceles that pose an infection and delayed complication risk.

## Introduction

The World Health Organization defines spinal cord injuries as partial or complete impairment of neurological performance (motor, autonomic, sensory and reflexes) resulting from spinal cord damage.^1^ These injuries are sustained either through blunt or penetrating trauma, of which blunt force trauma accounts for the majority of cases in spinal units.^2^

The most common causes of spinal cord injury, in developed countries, include falls and motor vehicle accidents.^3^ Penetrating spinal cord injuries (PSCIs) have a much lower incidence rate, of which penetrating stab wounds are most common.^4^ In the South African context, Sothmann et al. found the leading cause of spinal cord injuries to be secondary to motor vehicle accidents, while violence-related injuries (gunshot injuries and stab wounds) were less common; stab wounds contributing 8.6% of the total violence-related statistics.^5^ In a similar study conducted by Velmahos et al., between 1988 and 1992, it was found that penetrating spinal injury causes, including gunshot injuries and stab wounds, accounted for more than 50% of spinal cord injuries on a yearly basis.^6^ Similarly, Peacock et al. found 25% of spinal cord injuries to be secondary to penetrating causes (knives, bicycle spokes, axe, garden fork, sickle and sharpened broom stick, with knives most common).^2^

Throughout the literature, South African studies have the largest population-based cases of PSCIs,^2,7,8,9^ with the most frequent involved region being the thoracic spine, followed by the cervical spine.^2,10^ Jacobson et al.^10^ investigated the magnetic resonance imaging (MRI) findings in patients with PSCIs presenting to Groote Schuur Hospital between November 2004 and July 2005.^10^ Of the 22 patients, five patients (23%) were found to have extradural collections, of which only one had an extra-axial collection with mass effect on the spinal cord; however, because of the patient’s complete neurological deficit and associated spinal cord tract, surgical treatment was not deemed necessary. The most prevalent MRI findings in the study by Jacobson et al. were spinal cord signal changes and a perceived wound tract in the spinal cord.

Prior to the advent of MRI, many of these injuries were managed conservatively without imaging. Magnetic resonance imaging is the gold standard for evaluating the spinal cord, including ligamentous and other soft tissue structures, occult osseous injuries and disc material.^11,12^

In a review study by Peacock et al.,^2^ it was found that 80% of 450 penetrating injuries of the spinal cord resulted in incomplete neurological fallout, of which 4% of these patients underwent laminectomies for indications that included retained foreign objects and cerebrospinal fluid (CSF) leaks. Good post-operative outcomes were achieved to the extent that 66% of patients were able to walk either unassisted or with minimal support. This study was, however, conducted before MRI machines were commercially available.

Jacobson et al.^10^ found that conservative management often leads to partial neurological recovery; however, in the South African context, this has not always proven to be true as some cases of PSCIs may deteriorate without surgery.^2,8^ Conservative management may differ between institutions, however, because of penetrating injuries being considered contaminated wounds; antibacterial and tetanus prophylaxis are widely considered necessary.^10,13^ According to International and South African literature, indications for surgery include progressive neurological deficit, persistent CSF leaks, infection, foreign body and incomplete neurological deficit with a surgically correctable compressive lesion.^2,9,14^

The aim of this study was to compare our MRI-related imaging findings in patients with PSCI secondary to stab wounds with national and international literature and to determine in which percentage of these patients, MRI findings necessitated emergency spinal surgery.

## Research methods and designs

### Study design

The study was a retrospective, descriptive study.

### Research setting and sampling method

The Pelonomi Tertiary Hospital Trauma Unit (PTHTU) serves a large population in central South Africa, the Northern Cape and Lesotho. At our institution, urgent MRI was acquired by a Siemens Magneton Aera MRI 1.5 Tesla machine on the patients who presented with neurological fallout in the setting of acute spinal cord penetrating injury. This was performed to ascertain the need for emergent spinal surgery.

Permission to retrospectively access both the hospital information system (HIS) and the PTHTU trauma register was obtained from the hospital supervisor prior to the start of this study. All patient details were kept confidential and were only available for review by the current authors.

We included all patients who sustained PSCIs secondary to stab wounds and presented to the PTHTU during the time period of 01 August 2013–30 September 2016. A retrospective audit of the trauma unit patient registry was performed for this time and consecutive sampling made.

Patient information was then cross-referenced on the picture archiving and communication system (PACS) and those patients who underwent spinal MRI during this time period were selected and viewed. A total of 56 patients were included in the study. Seven patients were excluded from the study because of incomplete data entered onto the HIS, PTHTU trauma registry or patient files.

### Data collection

All included patient data were entered into a Microsoft Excel database. Captured information included patient demographics, site of the penetrating injury and MRI findings, whether the patient had complete or incomplete neurological deficit on admission and whether surgery was performed or not (Table 1).

**TABLE 1:** Magnetic resonance imaging findings in all stab wounds.

MRI findings	*N*	Yes		No	
		%	*n*	%	*n*
Soft tissue fluid collection	58	56.9	33	43.1	25
Intradural air	58	8.6	5	91.4	53
High signal wound tract on T2	58	96.6	56	3.4	2
Cord swelling or oedema	56†	82.1	46	17.9	10
Cord signal changes	56†	91.1	51	8.9	5
Extradural blood	58	34.5	20	65.5	38
Extradural CSF	58	19.0	11	81.0	47
Subdural blood	58	13.8	8	86.2	50
Retained objects	58	3.4	2	96.6	56

MRI, magnetic resonance imaging; CSF, cerebrospinal fluid.

† , Stabbed in the spinal canal below the level of the cord.

Neurological deficit was either classified as complete (total loss of sensory and motor function distal to the level of injury) or incomplete (neurological deficit with some residual motor or sensory function below the level of the injury). Brown-Sequard syndrome was diagnosed when there was ipsilateral paralysis with contralateral loss of spinothalamic function (pain and temperature sensation). Brown-Sequard plus (variants) were diagnosed when there were partial features of the typical Brown-Sequard syndrome, including asymmetric paresis and bilateral sensory disturbances.^15^

Magnetic resonance imaging sequences included a combination of T2-, T1-, short tau inversion recovery (STIR)-, gradient recalled echo (GRE)-, diffusion tensor imaging (DTI) sagittal, T2 coronal, selected T2 and GRE axial images. There was no set protocol for spinal penetrating injuries at that time. Magnetic resonance imaging sequences are summarised in Table 2.

**TABLE 2:** Magnetic resonance imaging sequences.

Sequences	%	*n*
Sag T2	94.6	53
Sag T1	82.1	46
Sag T2 STIR	91.1	51
Ax T2 GRE	80.4	45
Sag T2 GRE	30.4	17
Ax T2	42.9	24
Ax T1	21.4	12
Cor T2	19.6	11
Cor T2 STIR	33.9	19
DTI	50.0	28

Sag, sagittal; Ax, axial; GRE, gradient recalled echo; T1, spin-lattice relaxation time; T2, spin-spin relaxation time; Cor, coronal; STIR, short tau inversion recovery; DTI, diffusion tensor imaging.

The MR investigations were individually reviewed by the authors, one a fourth-year radiology fellow and the other a qualified diagnostic radiologist. All MRI findings were documented in the Excel database. Expansion of the cord was regarded as cord oedema, while linear cord signal changes were thought to represent a spinal cord laceration.

### Data analysis

Statistical analysis of all data was performed by the Department of Biostatistics at the University of the Free State. Results were summarised by frequencies and percentages for categorical variables and means, standard deviations or percentiles for numerical variables.

### Ethical consideration

Ethical clearance was obtained from the University of the Free State’s Health Sciences Research Ethics Committee (UFS-HSD2016/133), and permission was obtained from the Free State Provincial Health Research Committee (FS 2016RP12 860). All directly identifiable patient information (name, surname, date of birth and so on) were removed to ensure patient confidentiality.

## Results

The median age of the included 56 patients was 26.5 years, of which the interquartile range was 22.5–33.5 years. Fifty-eight penetrating spinal cord stab wounds were evaluated among the 56 patients. Two patients were presented with two penetrating spinal cord stab wounds. The sample of 56 patients consisted of eight female patients (13.8%) and 48 male patients (86.2%). The most frequent site of penetration was the thoracic spinal cord (*n* = 30, 53.6%), followed by the cervical spinal cord (*n* = 21, 37.5%) and lumbar spinal cord (*n* = 5, 8.9%).

The majority (69.6%) of patients received imaging during the first 24 h of presenting to the trauma unit. The remaining 30.4% of patients received delayed imaging (>24 h after admission) for variable reasons (delayed peripheral hospital referral, high patient burden, other occult injuries, and so on).

Incomplete neurological deficit was more frequent (85.4%), while 14.6% presented with complete neurological fallout. Brown-Sequard and related variants were reported in 42 (72.4%) of the 56 cases at discharge. Despite a normal neurological examination according to the assessment by the trauma physician in one study patient, a non-compressive extradural haematoma without obvious spinal cord signal changes was present. Documented MRI findings are summarised in Table 1.

**FIGURE 1:**
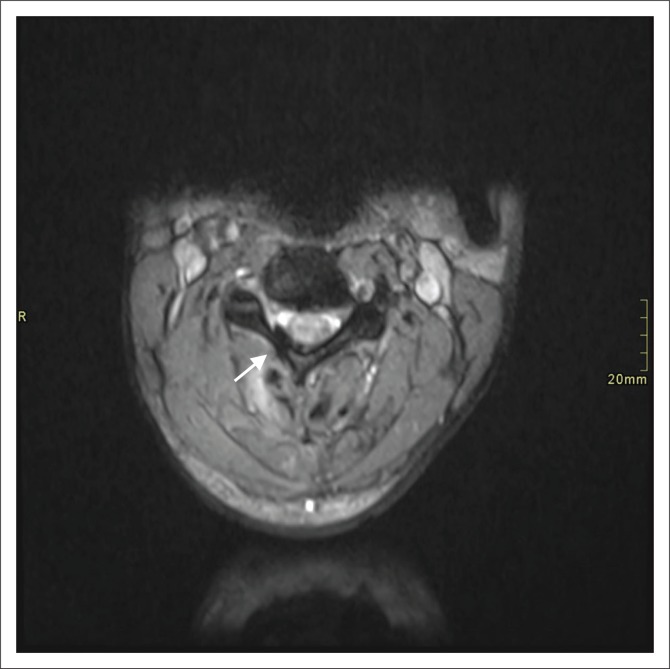
Axial gradient recalled echo image demonstrating subdural haemorrhage (arrow).

Table 2 displays the MRI protocols utilised. Sag T2 was included in the imaging protocol of 94.6% of patients, while Sag T2 STIR was performed in 91.1% of cases. A high T2 signal wound tract of the soft tissues was present in 96.6% of the cases in our study (Figure 1). Other common findings included linear or irregular spinal cord signal changes 91.1% (*n* = 51) and cord oedema 82.1% (*n* = 46). Intra-dural air was found in five cases (8.3%).

**FIGURE 2:**
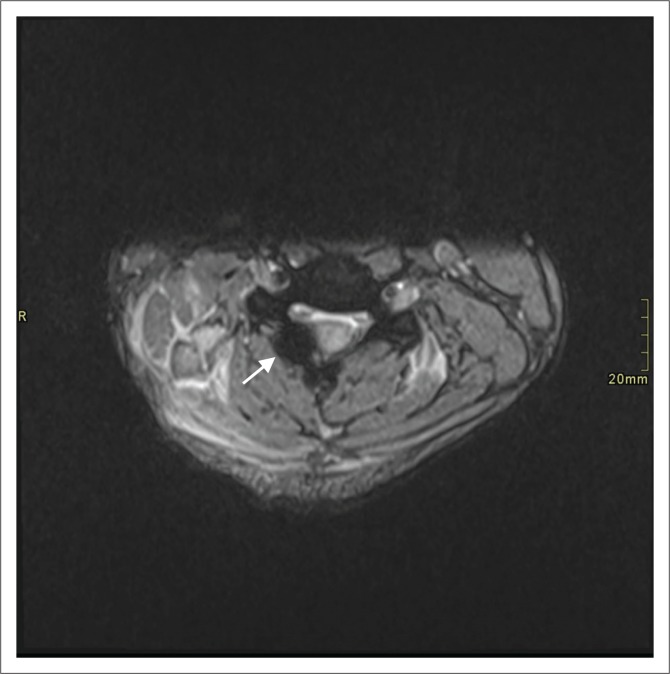
Axial gradient recalled echo image demonstrating extradural haemorrhage (arrow).

A total of 39 extra-axial collections were documented in 30 penetrating injuries. These were classified as extradural haemorrhage (*n* = 20), extradural CSF (*n* = 11) and subdural haemorrhage (*n* = 8) (Figures 1 and 2). Four patients (6.9%) demonstrated pseudo-meningoceles.

Spinal radiography was performed in 32.1% (*n* = 18) of the patients, while computed tomography (CT) was performed in 41.1% (*n* = 23). Metallic foreign material was noted in two of our study patients on MRI, of which one patient did not receive X-rays or a CT scan prior to the MRI.

Diffusion tensor imaging was performed in half of the study population (*n* = 28) (Table 2). Diffusion tensor imaging confirmed the diagnosis of spinal cord injury in 71.4% (*n* = 20) of these cases. Inconclusive results were present in five of the cases that received DTI – this was thought to be because of technical factors that included motion artefact, CSF flow and small calibre of the spinal cord.

Only one patient received emergent spinal surgery, despite no neurological deterioration. This consisted of emergency decompressive laminectomy and duroplasty, based on the suspicion of an extradural haematoma on MRI imaging. Upon reviewing the imaging for the purpose of this study, an extradural haematoma was not present. Nevertheless, the patient’s neurological status did not improve post-operatively and the patient was discharged for rehabilitation.

We recorded two mortalities that occurred after a period of admission, of which both presented with quadriplegia. Post-mortem reports could not be obtained.

## Discussion

Penetrating assault cases, including PSCIs, are a relatively common occurrence at PTHTU and in Southern Africa. Our study results are comparable to other studies conducted in South Africa by Peacock et al.^2^ and Jacobson et al.,^10^ with penetrating thoracic spinal cord injuries ranking highest, especially in young men. During the study period, PTHTU attended to 742 penetrating assault cases compared to 517 blunt assault cases.^16^

Controversy regarding the management of PSCI is still a reality. Studies have shown that conservative management often leads to partial neurological recovery without surgical intervention; however, some cases with missed spinal pathology may deteriorate without surgery.^2,8^ Indications for emergent surgery include progressive neurological symptoms, persistent CSF leaks and incomplete neurological deficit with a compressive lesion.^2,9,17^ If local infection (abscess formation) develops, surgical intervention may also be required as was found by Jacobson et al.^10^ None of our included study patients demonstrated neurological deterioration.

In patients with stable neurological symptoms, surgery is less likely to be performed, even in the presence of cord signal changes. This is because of the understanding that the cord injury is causing the neurology rather than the extra-axial collection causing the symptoms.^10^ However, in cases with isolated cord oedema in the presence of an extra-axial collection, surgery will likely be performed because of the suspicion that the collection is the cause of the neurology. In our study, only one patient was thought to have a compressive extradural haematoma. This patient, however, also demonstrated cord signal changes because of cord laceration and did not deteriorate neurologically. No neurological improvement was recorded post-surgery.

It is our opinion that the decision regarding surgery should thus be more influenced by neurological deterioration rather than the evidence of a compressive extra-axial haematoma on MRI in patients who also demonstrate cord signal changes.^2,8^ Patients who demonstrate no neurological deterioration should receive conservative management, consisting of a course of intravenous antibiotics, tetanus prophylaxis, as well as rehabilitation which includes physiotherapy and occupational therapy on a case-by-case basis.

In the included patients, none of the aforementioned indications for emergency spinal surgery, in the acute phase, was present on the MRI. In our experience, MRI in the acute phase setting did not significantly alter the surgical management of the patients.

Four patients (6.9%) did, however, demonstrate pseudo-meningoceles on review of the initial MRI studies. A persistent CSF leak is one of the indications for emergent surgery because of the risk of infection.^2,9,17^ These patients are also most likely to benefit from follow-up MRI imaging.

Migration of ferromagnetic components remains a concern with MRI.^18^ Prior to MRI, spinal X-rays and CT scans are helpful to evaluate the extent of bone injury, the penetrating wound trajectory, the presence of foreign material (especially metallic), bone fragments or air.^19^ Metallic foreign material was noted in two of our patients on MRI, of which one patient did not receive X-rays or a CT scan prior to the MRI. Although spinal radiography was performed in only 32.1% (*n* = 18) and CT was performed in only 41.1% (*n* = 23) of the patients, we suggest that all patients receive X-rays or CT prior to MRI to rule out the presence of metallic foreign material.

Patient prognosis in acute PSCI is best correlated with findings on T2-weighted sagittal MRI sequences.^11^ More recently, DTI has been used for prognostication purposes.^20^ Cord oedema, contusion and haemorrhage are best depicted on T2 sequences,^11^ while T2 GRE is more sensitive for demonstration of an acute haematoma.^19^

**FIGURE 3:**
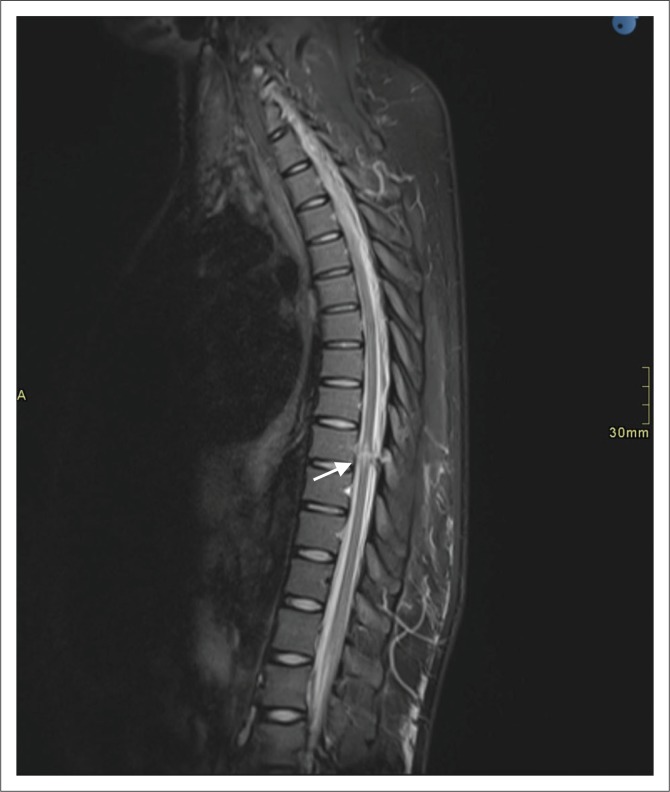
Sagittal T2 short tau inversion recovery image demonstrating high T2 signal wound tract, cord oedema and cord signal changes in the thoracic spine (arrow).

**FIGURE 4:**
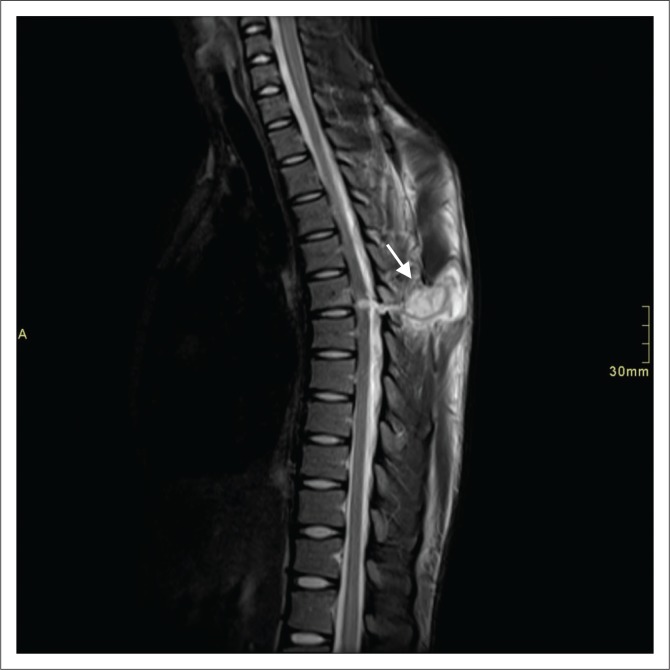
Sagittal T2 short tau inversion recovery image demonstrating pseudo-meningocele (arrow), high T2 signal wound tract, cord oedema and cord signal changes in the thoracic spine.

**FIGURE 5:**
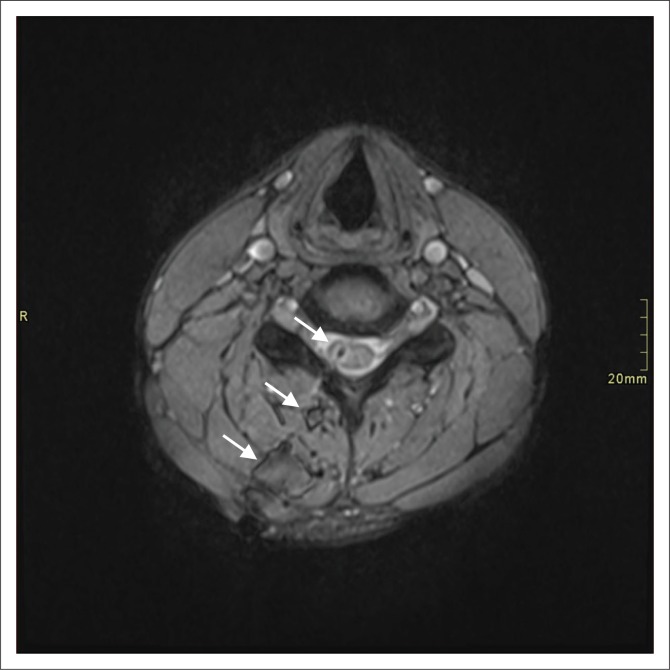
Axial gradient recalled echo image demonstrating cord blood and paraspinal muscle blood along the stab wound tract (arrows).

**FIGURE 6:**
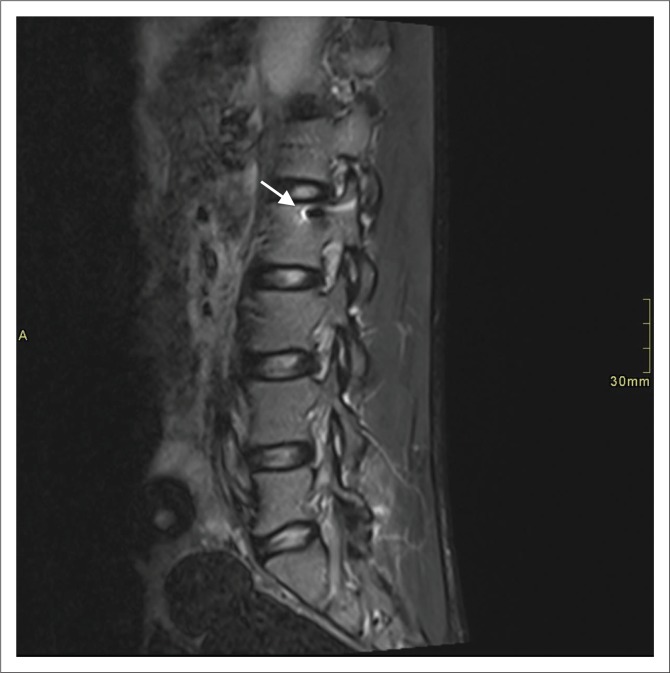
Sagital T2 short tau inversion recovery image demonstrating retained metallic foreign object in the L2 vertebral body.

In our study, the majority (94.6%) of the patients had T2-weighted sagittal imaging, while 91.1% of cases had sagittal T2 STIR included. Even though T1- and T2-axial imaging does not serve prognostication purposes,^11^ it does aid in deciding whether haematoma is intra- or extra-axial. In our experience, cord laceration was best depicted on T2 STIR sagittal images (Figure 3), while fluid collections were best seen on T2 STIR and T2 sagittal images (Figure 4). Gradient recalled echo images were the best at depicting cord haemorrhage (Figure 5) although without spinal X-rays and CT, there is difficulty in differentiating intra-spinal air and foreign fragments (Figure 6) from haemorrhage.

One of the limitations of conventional MRI is the evaluation of the white matter tract integrity.^21^ Diffusion tensor imaging is an imaging modality that was first described by Basser et al. in 1994 and has superior performance in visualising microstructures compared to other MRI sequences (i.e. T1- and T2-weighted images). This imaging sequence is acquired based on the direction of water molecule diffusion in tissues which is limited by structures such as the myelin sheath and cell membrane. Anisotropy is the movement of water molecules in one direction. Disruption of white matter tracts leads to a lowered fractional anisotropy (FA) that is sensitive for microstructural changes. The location and severity of spinal cord injury can therefore be determined using DTI, which is much more sensitive for PSCI than conventional MRI. It has been found that the findings on DTI correlate with histologic axonal injury, as well as functional recovery.^20^ According to Xiao-Hui Li, FA values were lowest at 24 h after injury, even though at 6 h post-injury, a significant decrease in FA could already be seen. The optimal timing to perform DTI was thus concluded to be at 24 h post-injury.^22^

**FIGURE 7:**
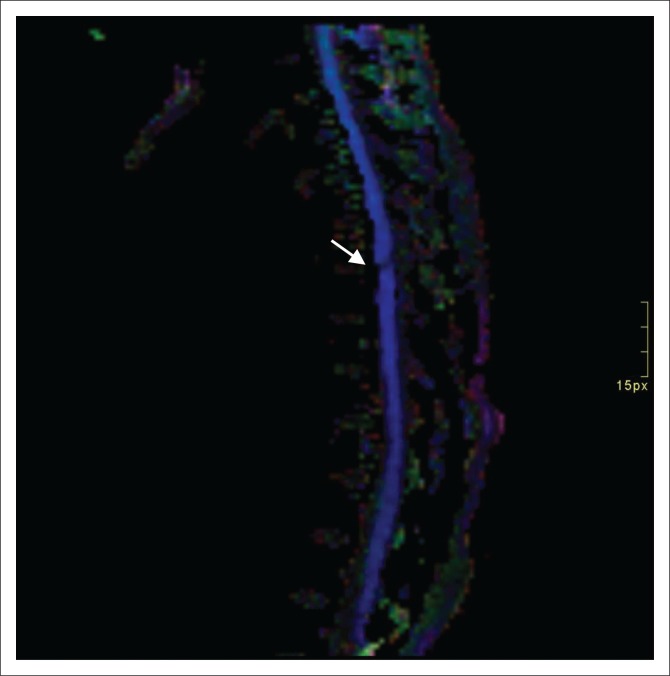
Sagittal diffusion tensor imaging of the thoracic spine demonstrates disrupted white matter tracts with decreased fractional anisotropy (arrow).

**FIGURE 8:**
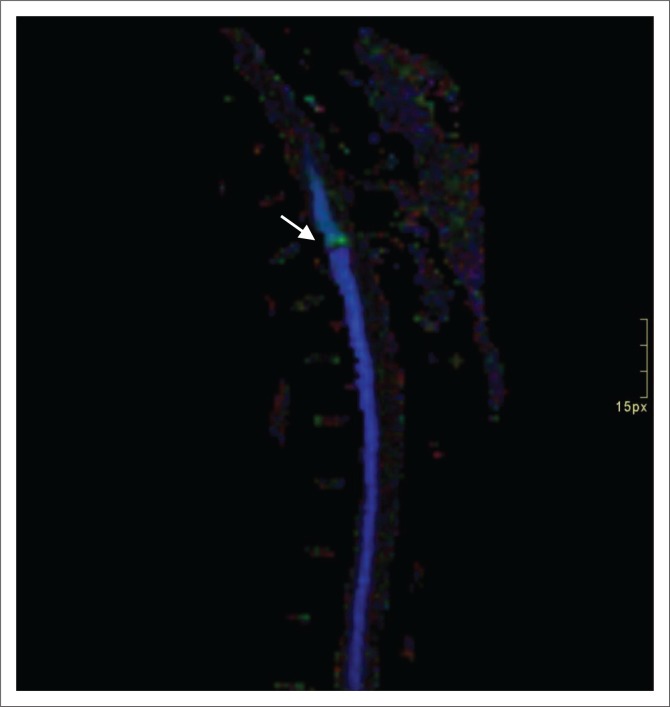
Sagittal diffusion tensor imaging of the thoracic spine demonstrates disrupted white matter tracts with decreased fractional anisotropy (arrow).

Diffusion tensor imaging was not routinely performed on MRI investigations during the time the study patients received imaging. It was, however, included in some cases, but since no set protocol for imaging existed, only half of the study patients received DTI. Disrupted white matter tracts (Figures 7 and 8), as evidenced by decreased FA, was demonstrated in 72.4% of cases. The small calibre of the spinal cord, CSF flow, patient motion and susceptibility-weighted artefacts are all reasons described in DTI-related studies that may have led to inconclusive findings in five of our study patients.^23,24^

All the patients who demonstrated decreased FA in our study also had cord signal changes (cord blood and high signal linear cord tract changes), whereas all the study patients who had no change in FA demonstrated no cord signal changes.

In a literature review performed by Goulet et al., it was found that most studies conducted MRI imaging within 72 h following a PSCI, even though no specific guidelines exist pertaining to the best timing of MRI after PSCI.^11^ More than 70% of our patients received imaging within 24 h of presentation to hospital. In our experience, although the timing did not alter the acute management of our study patients, it does seem to be beneficial for demonstration of axonal disruption and therefore clinical outcome, compared to imaging performed after 24 h. This also demonstrated that PTHTU imaging timing protocols were comparable to international practices.

From our study, it was evident that a set protocol regarding imaging sequences should be in place if future studies are to be conducted on the prognostication evaluation of MRI for PSCI in our hospital setting. A universal neurological evaluation score should be applied. We suggest that the best imaging protocol for these studies should include T1-, T2-, and T2 STIR-, GRE sagittal, T1-, T2- and GRE axial, as well as DTI within a 24-h period. If pseudo-meningoceles are demonstrated on initial MRI, follow-up imaging should be performed during the next two weeks for CSF leak persistence.

## Study limitations

Clinical notes regarding the neurological evaluation and management were retrospectively accessed and in some cases were suboptimal or non-retrievable. Neurological evaluation was not documented according to the American Spinal Injury Association (ASIA) or similar universal scoring systems, possibly limiting the use of these study results for further studies.

Magnetic resonance imaging was a novel imaging method at the Pelonomi Tertiary Hospital radiology unit (PTHRD) during the time of the study. There was no set protocol for imaging of PSCIs at that time, and therefore, all patients did not receive exactly the same MRI imaging sequences.

All patients did not receive an MRI investigation within the same time frame from admission because of multiple factors (high and variable patient burden, peripheral patient referrals with patient transfer delays, and so on). All patients were thus not imaged within the acute phase post-PSCI (24 h after injury).

Although our study included 56 patients, the study population is still relatively small. Only two reviewers were used to evaluate the MRI findings. One of the reviewers was a qualified radiologist, while the other was a fellow in training. The results of MRI findings could therefore be slightly skewed because of difference in experience with interpretation of MRI investigations.

### Future recommendations

We suggest that a prospective follow-up study should be conducted, where a universal clinical scoring system is used and MRI investigations are performed within 24 h of admission, using a set protocol regarding sequences that could also include DTI.

## Conclusion

Although our MRI findings did not alter the surgical course of action, MRI in the post-traumatic phase (<24 h) did detect the presence of a pseudo-meningocele, which posed a possible infection risk.

Both South African and international literature support the need for an MRI in PSCIs. This is especially important in cases where certain pathology, which warrants emergent surgery, might be missed on alternative imaging modalities and ultimately lead to a poorer prognosis. Surgical management versus conservative management in such cases is still debated, but relative indications for surgical intervention are stated in international and South African literature^9,10,13^

At our institution, the burden of assault-related penetrating injuries, including spinal cord penetrating injuries, is high. Spinal cord injuries not only contribute to the financial burden of the health system, but also have a tremendous effect on the physical and mental well-being of patients and their social support structures. Optimising the neurological outcome of these patients is essential and requires a multidisciplinary approach, including but not limited to radiological services that guide management of these cases.

Although management of patients with PSCI should be guided largely by the neurological status and the deterioration thereof, rather than only MRI findings, MRI in the acute phase (24 h) should be considered as part of a set imaging protocol in such patients. This will aid in the diagnosis of white matter tract disruption and prognostication of these patients. We also suggest that in cases where pseudo-meningocele is demonstrated, follow-up MRI investigation should be performed to evaluate the progression or persistence of the CSF leak.
